# Circulating osteogenic progenitors and osteoclast precursors are associated with long-term glycemic control, sex steroids, and visceral adipose tissue in men with type 2 diabetes mellitus

**DOI:** 10.3389/fendo.2022.936159

**Published:** 2022-09-12

**Authors:** Elliot Ballato, Fnu Deepika, Mia Prado, Vittoria Russo, Virginia Fuenmayor, Siresha Bathina, Dennis T. Villareal, Clifford Qualls, Reina Armamento-Villareal

**Affiliations:** ^1^ Division of Endocrinology, Diabetes and Metabolism, Department of Medicine, Baylor College of Medicine, Houston, TX, United States; ^2^ Center for Translational Research on Inflammatory Disease, Michael E DeBakey Veterans Affairs (VA) Medical Center, Houston, TX, United States; ^3^ Biomedical Research Institute of New Mexico, Albuquerque, NM, United States; ^4^ Research Service Line, New Mexico Veterans Affairs Health Care System, Albuquerque, NM, United States

**Keywords:** osteoblast (OB), osteoclast (OC), body composition, type 2 diabetes mellitus, circulating osteogenic progenitors

## Abstract

**Introduction:**

Type 2 diabetes mellitus (T2DM) is well-known to be associated with normal bone density but, concurrently, low bone turnover and increased risk for fracture. One of the proposed mechanisms is possible derangement in bone precursor cells, which could be represented by deficiencies in circulating osteogenic progenitor (COP) cells and osteoclast precursors (OCP). The objective of our study is to understand whether extent of glycemic control has an impact on these cells, and to identify other factors that may as well.

**Methods:**

This was a secondary analysis of baseline data from 51 male participants, aged 37-65 in an ongoing clinical trial at Michael E. DeBakey VA Medical Center, Houston, Texas, USA. At study entry serum Hemoglobin A1c was measured by high-performance liquid chromatography osteocalcin (OCN) and C-terminal telopeptide of type 1 collagen (CTx) were measured by ELISA, and testosterone and estradiol by liquid-chromatography/mass-spectrometry. Areal bone mineral density (BMD), trabecular bone score and body composition were measured by dual energy x-ray absorptiometry, while COP and OCP were measured by flow cytometry.

**Results:**

When adjusted for serum testosterone, parathyroid hormone, and 25-hydroxyvitamin D, those with poor long-term glycemic control had significantly higher percentage of COP (p = 0.04). COP correlated positively with visceral adipose tissue (VAT) volume (r = 0.37, p = 0.01) and negatively with free testosterone (r = -0.28, p = 0.05) and OCN (r = -0.28, p = 0.07), although only borderline for the latter. OCP correlated positively with age, FSH, lumbar spine BMD, and COP levels, and negatively with glucose, triglycerides, and free estradiol. Multivariable regression analyses revealed that, in addition to being predictors for each other, another independent predictor for COP was VAT volume while age, glucose, and vitamin D for OCP.

**Conclusion:**

Our results suggest that high COP could be a marker of poor metabolic control. However, given the complex nature and the multitude of factors influencing osteoblastogenesis/adipogenesis, it is possible that the increase in COP is a physiologic response of the bone marrow to increased osteoblast apoptosis from poor glycemic control. Alternatively, it is also likely that a metabolically unhealthy profile may retard the development of osteogenic precursors to fully mature osteoblastic cells.

## Introduction

A host of factors can lead to disorders of bone remodeling including but not limited to aging, menopause, hormonal imbalance, vitamin D deficiency, medications, immobilization, and chronic kidney disease, among others (1). Recently, type 2 diabetes mellitus (T2DM) has been added to this list. Our group previously reported that men with T2DM with or without hypogonadism had reduced bone turnover markers (2). This finding is in agreement with observations from other investigators suggesting impaired bone remodeling in these patients (3–6). Furthermore, our group also showed a differential effect of glycemic control on bone turnover. We recently reported that men with poor blood glucose control, defined as hemoglobin A1c (A1c) of ≥7% had suppressed bone turnover relative to men with good glycemic control (defined as A1c of <7%) (7). In fact, the bone turnover markers of the latter were comparable to patients without T2DM. These glycemia-associated differences in bone remodeling are in turn reflected by differences in bone microarchitecture and strength, with poor indices among those with poor control (8).

Diabetes mellitus is associated with accumulation of advanced glycation end products (AGE’s) and non-enzymatic glycation products (NEG’s) which can result in microstructural defects in bone (9, 10). Given the reduced bone remodeling in these patients, it is likely that repair of these microstructural damages and replacement of old with new bone is impaired, leading to skeletal fragility. The underlying mechanism for this observed reduction in bone remodeling in patients with T2DM is not well-established. Nevertheless, there are suggestions that low bone turnover in the context of T2DM is mostly a problem of bone formation since hyperglycemia impairs osteoblastogenesis (11, 12) and could be the initiating factor for reduced bone remodeling given the cross-talk between osteoblasts and osteoclasts. One study reported lower osteoblastic progenitors in patients with T2DM (13). However, it remains unclear if the degree of glycemic control affects the proliferation and differentiation of these cells *in-vivo*. The objective of this study is to evaluate the effect of glycemic control on circulating osteogenic progenitor (COP) cells and osteoclast precursors (OCP) in the circulation. Secondarily, we will also evaluate the effect of anthropometric, hormonal and metabolic factors on circulating COP and OCP.

We hypothesize that poor glycemic control (ie A1c>7%) is associated with fewer circulating COP and OCPs. Furthermore, we also hypothesize that other factors (hormonal and metabolic) will affect the flux of these cells in circulation resulting in alterations in bone parameters. To our knowledge, this is the first study of its kind examining the effect of glycemic control on COP and OCPs levels in adult males with T2DM.

## Materials and methods

### Patient population

This is a secondary analysis of baseline data from the study “Testosterone Therapy and Bone Quality in Men With Type 2 Diabetes Mellitus and Hypogonadism” which is ongoing at the Michael E DeBakey VA Medical Center since October 2019 (NCT03887936). The inclusion/exclusion criteria for this study are as described previously (14), but briefly, male veterans, 35 to 65 years old, with an average fasting morning total testosterone (T) level from 2 measurements of <300 ng/dL taken at least a day apart and symptoms of hypogonadism as assessed by quantitative Androgen Deficiency in the Aging Male survey (qADAM) (15), having T2DM of <15 years duration with an A1c of <10.5% and body mass index (BMI) <35 kg/m^2^. Diagnosis of T2DM was by chart review and A1c measurement at study entry, using widely-accepted diagnostic criteria of A1c≥6.5% and fasting plasma glucose>125mg/dL or use of antidiabetic medications (16). Excluded were those with 1) a history of prostate or breast cancer, 2) testicular disease, 3) untreated severe sleep apnea, 4) any illness that could prevent the subject from completing the study or diseases that interfere with bone metabolism 5) hematocrit of >50%, 6) prostate-related findings on digital rectal exam, 7) serum PSA of ≥4.0 ng/mL or ≥3.0 ng/mL for African-Americans, 8) International Prostate Symptom Score (IPSS) > 19, 9) on androgen therapy, or selective androgen receptor modulators, 10) on medications that affect bone metabolism, 11) current alcohol use of >3 drinks/day, 12) history of deep vein thrombosis, pulmonary embolism, stroke or recent diagnosis of coronary artery disease 13) a T-score ≤−2.5 assessed by dual-energy x-ray absorptiometry at the lumbar spine, total femur or femoral neck, or a history of fragility fractures (spine, hip or wrist), and/or 14) fasting total T less than 50 ng/dL.

### Body mass index

Body weight and height were measured by standard weighing scale and stadiometer, respectively. BMI (kg/m^2^) was calculated by dividing the weight (in kilograms) by height (in meters) squared.

### Biochemical analyses

Blood was obtained in the morning after an overnight fast, processed, and samples were stored at -80°C until analysis. Serum total T and estradiol were measured by liquid chromatography/mass spectroscopy by LabCorp laboratory (Burlington, NC, USA), total T intra-assay CVs are 7.4%, 6.1%, 9.0%, 2.3%, and 0.9% at 0.65, 4.3, 48, 118, and 832 ng/dL, respectively. Inter-assay CVs are 8.9%, 6.9%, 4.0%, 3.6%, and 3.5% at 0.69, 4.3, 45, 117, and 841 ng/dL, respectively. The detection range is 0.5 to 2,000 ng/dL (17). Estradiol assay sensitivity is 0.23 to 405 pg/mL, intra-assay CV is 1.4% to 11.8% and inter-assay CV is 4.8% to 10.8% (2). SHBG was measured with electrochemiluminescence immunoassay by LabCorp laboratory (Burlington, NC, USA). The following were measured by the clinical laboratory at the Michael E. DeBakey VA Medical Center: A1c was measured by high-performance liquid chromatography using Tosoh Automated Glycohemoglobin Analyzer HLC-723G8. (Tosoh Bioscience, Inc. South San Francisco, CA, USA); triglycerides were measured by fluorometric assay and LDL and HDL were measured by colorimetric assay by UNICEL DxC (Beckman Coulter, Inc., 250 S. Kraemer Blvd., Brea, CA 92821 USA). Detection limits for these measurements are: 11–500 mg/dl for LDL, 5–135 mg/dL (0.13–3.5 nmol/L) for HDL, 10–1,000 mg/dL (0.1–11.3 mmol/L) for triglycerides; CVs <10% for all measurements (17). The free T index was calculated by using the formula previously described by Sowers et al. (18) i.e. 100×T (ng/dl)/28.84×SHBG (nM) which is unit free. The free E_2_ index (FEI) was calculated as the molar ratio of total E_2_ to SHBG (pmol/nmol) (19). Fasting glucose was measured using Unicel *DxC 800* Auto-analyzer (Beckman Coulter, Fullerton, CA, USA). The following were measured using enzyme-linked immunosorbent assay kits: CTx, marker of bone resorption (Crosslaps; Immunodiagnostic System Inc., Gaithersburg, MD); and OCN, marker of bone formation, (Metra OC; Quidel Corporation, San Diego, CA); and high-sensitivity C-reactive protein (hs-CRP) (Eagle Biosciences, Inc., Nashua, NH). The coefficients of variation (CVs) for the above assays in our laboratory are <10% and <3.5% for A1c.

### Mean -12M A1c

Mean A1c was obtained from medical record review of each patient’s chart using the Veterans Affairs Computerized Patient Record System, A1c values measured between 9 and 15 months prior to study enrollment. For each participant, the average of these A1c measurements was calculated to give a single 12-month average A1c value, the -12M A1c.

### Flow cytometry

Identifying COP and OCP can and has been done by flow cytometry. OCP express several identifying proteins, chiefly among them M-CSF’s receptor MCSFR. Gossiel et al. have previously described a staining protocol where dual CD14CD11b+, CD14MCSFR+, CD14CD120b+ cells are identified as OCPs (20). CD14 is a cell-surface receptor in monocytes that responds to lipopolysaccharides and serves as a pattern recognition receptor (21, 22), CD11b is an integrin protein found on many leukocytes of the macrophage lineage (23), and CD120b, also known as TNFR2, is a surface receptor for TNF-α. Osteoblasts and circulating COP are identified chiefly with anti-OCN antibodies as described by others (13, 24–26). To exclude B, T, and NK cells, the CD3-CD19-CD56- cell population can be gated. Finally, the fluorescence minus one technique, as described by Perfetto et al. (27), can be useful in ruling out background noise to identify COP.

**Figure 1:**
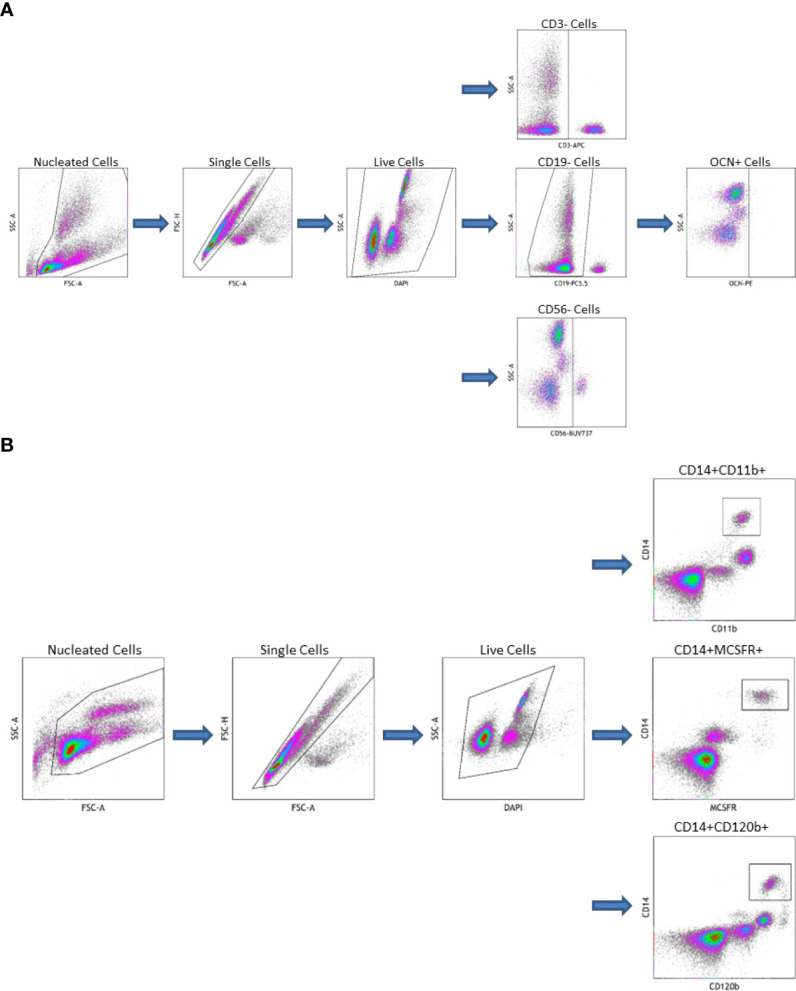
**(A)** Gating Strategy for Circulating Osteogenic Progenitor Cell Isolation. Cells are first sorted along forward/side scatter to identufy nucleated cells, then forward area and height to identify singlets, then DAPI-negative cells are identified as viable. Cells that are CD3-CD19-CD56-are gated from this population the OCN+ cells are identified as osteoblast progenitors. **(B)** Gating Strategy for Osteoclast Precursor Cell Isolation. Cells are first sorted along forward/side scatter to idfentify nucleated cells, then forward area and height to identify singlets, then DAPI-negative cells are identified as viable. Cells that are CD14+CD11b+, CD14+MCSFR+, or CD14+CD120b+ are identified as OC precursors.

OCN positive COP were isolated from peripheral blood using a previously described method with modification (13, 24, 25, 28). Peripheral blood mononuclear cells (PBMC) were isolated *via* Ficoll-Paque density gradient. Fresh, whole blood, treated with anticoagulant, was diluted 1:1 with phosphate-buffered saline, then layered on top of Ficoll-Paque density gradient at a ratio of 4:3. This was centrifuged for 35 minutes at 400 x g. The pellet was then isolated and washed twice with PBS. PBMC were then stored on ice for the remainder of the experiment. They were stained with a) combination of CD3 (BD Pharmingen, San Diego, CA, USA), CD19 (Beckman Coulter, Indianapolis, IN, USA), CD56 (BD Horizon, San Diego, CA, USA), OCN (Santa Cruz Biotechnology, Dallas, TX, USA), and 4′,6-diamidino-2-phenylindole (DAPI, Beckman Coulter, Indianapolis, IN, USA) b) just CD3, CD19, CD56, and DAPI (reflecting the fluorescence minus one technique (27)) or c) just DAPI. Viable cells that were OCN+ and CD3CD19CD56- were considered COP (see **Figure 1A**).

OCPs were also isolated from peripheral blood. First, PBMC were extracted from whole blood as described above. These were then stained with a) combination of CD14 (BD Pharmingen, San Diego, CA, USA), CD11b (ThermoFisher Scientific, Waltham, MA, USA), and DAPI b) CD14, CD11b, MCSFR (Abcam, Waltham, MA, USA), and DAPI c) CD14, CD11b, CD120b (BD Pharmingen, San Diego, CA, USA), and DAPI or d) just DAPI. Viable cells that were dual CD14CD11b+, CD14MCSFR+, or CD14CD120b+ were considered OCPs (see **Figure 1B**). All flow cytometry data was collected on a CytoFlex LX Flow Cytometer (Beckman Coulter, Indianapolis, IN, USA) and analyzed using Kaluza Analysis Software (Beckman Coulter, Indianapolis, IN, USA).

### Imaging studies

#### Areal BMD, trabecular bone score and body composition

aBMD was assessed by dual energy X-ray absorptiometry (DXA) on the lumbar spine, left proximal femur (right femur if history of prior surgery) for total femur and femoral neck regions of interest, and whole body using Hologic Discovery (Hologic Inc, Bedford, MA, USA). The CVs at our center are ~1.1% for the lumbar spine and ~1.2% for the proximal femur (19, 29). TBS of the spine images (using L1-L4) obtained by DXA was assessed using the TBS Insight 2.2 software (Med-Imaps, Merignac, France). TBS is a gray-level textural assessment calculated from the standard DXA spine images which is considered a measure of skeletal microarchitecture at the spine (30).

Measurement of body composition was performed by DXA (Hologic-Discovery; Enhanced Whole Body 11.2 software version; Hologic Inc, Bedford, MA; USA). Images were analyzed according to manufacturer’s instructions. The CV for fat mass and lean mass measurements in our center is 1.5% (19). Visceral adipose tissue (VAT) volume (g/cm^2^) was calculated from the DXA body composition scan using APEX software (version 5.5.2; Hologic Inc., Bedford, MA) as previously described (29).

### Statistical analyses

Results are presented as means ± standard deviation (SD) in the tables. A p-value <0.05 was considered significant, and <0.10 was considered borderline significant. Participants were grouped according to study entry and mean A1C levels, i.e. ≤7% and >7%. Group comparisons were performed by one-way analysis of covariance. The associations between COP and OCP cells with hormonal, metabolic and bone parameters were analyzed by simple correlation analysis. Independent predictors for each of these circulating cellular components were identified by multivariable regression analyses. This was performed by first identifying all variables that were significantly associated with the parameter of interest as candidate variables, then performing a backwards stepwise regression with all candidate variables included in the model. Significant predictors were identified and tabulated. Data were managed using Excel 2013 (Microsoft, Redmond, WA), and analyzed by Statgraphics Centurion XVI X64 (Statgraphics Technologies, Inc., The Plains, VA, USA).

## Results

### Demographics and medications

The data from 51 consecutive men who were able to provide the outcomes of interest were included in this analysis. Ages ranged from 37 to 65 with an average of 55.2 ± 6.3 years. Of the 51 men, 20 (39%) were African-American, 24 (47%) were non-Hispanic white, 6 (12%) were Hispanic, and 1 (2%) was Asian. Total T of the overall population was 265.0 ± 84.3 ng/dL, 43 participants were hypogonadal with an average Total T of 236.8 ± 44.4 while 8 were eugonadal with an average Total T of 328.4 ± 82.8 ng/dl. Average BMI was 31.7 ± 3.5. All participants had T2DM with average A1c 8.1 ± 1.4%. Mean duration of T2DM was 7.6 ± 5.7 years (see **Table 1**). Of the 51 participants, 42 were on medications for T2DM while 9 were not. Of the 42 participants on medications, 8 were on metformin alone, 2 on insulin alone, 4 on metformin and insulin in combination, and the other 28 were on different combinations of metformin, insulin, dipeptidyl peptidase 4 inhibitors, glucagon-like peptide-1 receptor agonists, sodium-glucose cotransporter-2 inhibitors, sulfonylureas, thiazolidinediones, and alpha-glucosidase inhibitors (see **Supplemental Table 1**).

**Table 1:** Demographic and clinical data of the study participants.

Parameter	Result (n = 51)
Age (years)	55.2 ± 6.3
BMI	31.7 ± 3.5
A1c at study entry (%)	8.1 ± 1.4
Testosterone (ng/dL)	265.0 ± 84.3
Duration of T2DM (years)	7.6 ± 5.7
Total Body Fat (%)	34.3 ± 4.3

Results are expressed as mean ± standard deviation. BMI, body mass index; A1c, glycated hemoglobin; T2DM, type 2 diabetes mellitus.

### Effect of glycemic control

To determine the effect of short- and long-term glycemic control, we divided our subjects according to A1c at study entry and the Mean (-12M) A1c into ≤ 7% (n = 15, good T2DM control) and >7% (n = 36, poor control), but only 46 subjects have data on COP and OCPs (good control N=15, poor control N=31). For clinical characteristics of subjects according to A1c at entry and Mean -12M A1c, please see **Supplementary Tables 2, 3**. Analysis according to A1c at study entry showed that poorly-controlled subjects had significantly longer duration of T2DM (9.0 ± 5.9 years vs 5.0 ± 4.5 years, p = 0.02), higher average A1c for the prior year (9.0 ± 1.9% vs 6.8 ± 1.0%, p < 0.001), lower 25-hydroxyvitamin D (23.8 ± 11.3 ng/mL vs 33.1 ± 14.7 ng/mL, p = 0.02), lower SHBG (22.0 ± 12.6 nmol/L vs 30.5 ± 11.3 nmol/L p = 0.02), and lower BMD at the total hip (1.062 ± 0.16 g/cm^2^ vs 1.159 ± 0.16 g/cm^2^, p = 0.05) (**Supplementary Table 2**). The significance of the latter disappears with adjustments for age and BMI (p = 0.14). Analysis according to Mean -12M A1c similarly showed that those with poor long-term glycemic control had significantly longer duration of T2DM (8.9 ± 5.9 years vs 4.7 ± 4.1 years p = 0.02). Furthermore, they had significantly lower total lean mass (63.1 ± 8.1 kg vs 68.2 ± 5.1 kg, p = 0.04) compared to those with good long-term glycemic control but significance was lost after adjustment for age and BMI, p = 0.20 (**Supplementary Table 3**). A separate analysis for BMD and body composition adjusted for age and BMI in both **Supplementary Tables 1, 2** was done showing no significant between-group differences in any of these parameters in the short- or long-term glycemic control categories.

The average COP in the entire population was 0.41 ± 0.13% and within the reference range of normal for COP (OCN+) reported in a prior study (31). Analysis of precursor cells in **Table 2** showed that those with long-term poor glycemic control had significantly increased percentage of COP when adjusted for factors that influence production, proliferation and differentiation of these cells such as PTH, 25-hydroxyvitamin D and free T levels (0.42 ± 0.12% vs 0.35 ± 0.11%, p = 0.04). However, when analysis was also adjusted for the duration of T2DM, the difference between the 2 groups became borderline (p=0.07). There were no significant differences in circulating OCP cells according to short- and long-term glycemic control.

**Table 2:** Circulating osteogenic progenitors and osteoclast precursors according to A1C at study entry and mean -12M A1c.

	A1c ≤ 7% (n = 15)	A1c >7% (n = 31)	P-value	Adjusted P*
A1C at study entry				
COP (%)	0.39 ± 0.15	0.42 ± 0.13	0.51	0.35
CD14CD11b+ (%)	4.20 ± 2.01	3.73 ± 1.91	0.45	0.77
CD14MCSFR+ (%)	3.95 ± 1.70	3.50 ± 1.87	0.44	0.81
CD14CD120b (%)	4.41 ± 2.15	3.83 ± 2.08	0.38	0.73
		Mean A1C		
COP (%)	0.35 ± 0.11	0.42 ± 0.12	0.10	**0.04**
CD14CD11b+ (%)	3.70 ± 2.35	3.93 ± 1.83	0.74	0.17
CD14MCSFR+ (%)	3.52 ± 2.03	3.69 ± 1.78	0.79	0.20
CD14CD120b (%)	3.80 ± 2.42	4.05 ± 2.02	0.73	0.12

Values are means ± SD, Bolded p-values are statistically significant. A1c, glycated hemoglobin; COP, circulating osteogenic progenitor. *Adjusted for free testosterone, 25-hydroxyvitamin D and parathyroid hormone.

### Association between COP and OCPs with hormonal and metabolic factors

COP were inversely correlated with serum free T (r = -0.28, p = 0.05) and OCN (r = -0.28, p = 0.07), although significance for the latter was only borderline **(** **Table 3**). OCPs, CD14CD11b+, CD14MCSFR+, and CD14CD120b+ cells positively correlated with age and negatively correlated with plasma glucose, and triglycerides. FSH was positively correlated with all three of the OCP populations, but LH was not significantly associated with any of them. The CD14MCSFR+ negatively correlated with free estradiol (r = -0.34, p = 0.03). The CD14CD120b+ population was positively correlated with serum 25-hydroxyvitamin D (r = 0.33, p = 0.03), while significance was borderline for the other two. COP also positively correlated with all OCPs (r = 0.34, p = 0.02, r = 0.38, p = 0.01, r = 0.33, p = 0.03 for CD14CD11b+, CD14MCSFR+, and CD14CD120b+ respectively). There were no correlations between short- and long-term A1c and any cellular parameters.

**Table 3:** Correlation analysis of circulating osteogenic progenitors and osteoclast precursors on hormonal and metabolic parameters of interest.

Parameter	COP (n = 46)		CD14CD11b+(n = 46)		CD14MCSFR+(n = 45)		CD14CD120b (n = 46)	
	r	(p)	r	(p)	r	(p)	r	(p)
Age (years)	0.11	(0.47)	0.33	**(0.02)**	0.39	**(0.009)**	0.32	**(0.03)**
Glucose (mg/dL)	-0.08	(0.60)	-0.32	**(0.03)**	-0.35	**(0.02)**	-0.32	**(0.03)**
LDL (mg/dL)	0.09	(0.54)	-0.27	(0.07)	-0.26	(0.08)	-0.25	(0.10)
HDL (mg/dL)	-0.01	(0.97)	-0.20	(0.19)	-0.14	(0.37)	-0.20	(0.19)
Triglycerides (mg/dL)	-0.20	(0.19)	-0.32	**(0.03)**	-0.35	**(0.02)**	-0.29	**(0.05)**
25-OHD (ng/mL)	0.07	(0.63)	0.28	(0.06)	0.28	(0.06)	0.33	**(0.03)**
LH (mIU/mL)	-0.02	(0.89)	0.11	(0.46)	-0.03	(0.83)	0.11	(0.47)
FSH (mIU/mL)	0.13	(0.39)	0.31	**(0.03)**	0.29	**(0.05)**	0.31	**(0.03)**
Free T index	-0.28	**(0.05)**	-0.24	(0.10)	-0.22	(0.14)	-0.22	(0.14)
Free Estradiol (pmol/nmol)	-0.17	(0.27)	-0.28	(0.06)	-0.34	**(0.03)**	-0.21	(0.18)
PTH (pg/mL)	-0.24	(0.11)	0.01	(0.92)	-0.03	(0.83)	0.11	(0.47)
OCN (ng/mL)	-0.28	(0.07)	0.01	(0.93)	0.02	(0.92)	0.01	(0.95)
CTX (ng/mL)	-0.22	(0.17)	-0.09	(0.60)	-0.08	(0.63	-0.15	(0.37)
Hs-CRP (mg/L)	0.04	(0.78)	-0.03	(0.84)	0.01	(0.94)	-0.003	(0.98)
A1c at study entry (%)	-0.09	(0.56)	-0.22	(0.14)	-0.22	(0.14)	-0.24	(0.11)
Mean -12M A1c (%)	-0.07	(0.71)	-0.23	(0.13)	-0.22	(0.14)	-0.25	(0.098)
COP (%)	–	–	0.34	**(0.02)**	0.38	**(0.01)**	0.33	**(0.03)**

Bolded p-values are statistically significant. COP, circulating osteogenic progenitors; LDL, low-density lipoprotein; HDL, high-density lipoprotein; 25-OHD, 25-hydroxyvitamin D; LH, leutinizing hormone; FSH, follicle stimulating hormone; Free T, serum free testosterone; PTH, parathyroid hormone; OCN, osteocalcin; CTX, C-telopeptide of type I collagen; Mean -12M, average of all A1c measurements between 9 and 15 months prior to study entry; hs-CRP, high-sensitivity C-reactive protein.

### Association between COP and OCPs with body composition

**Figure 2:**
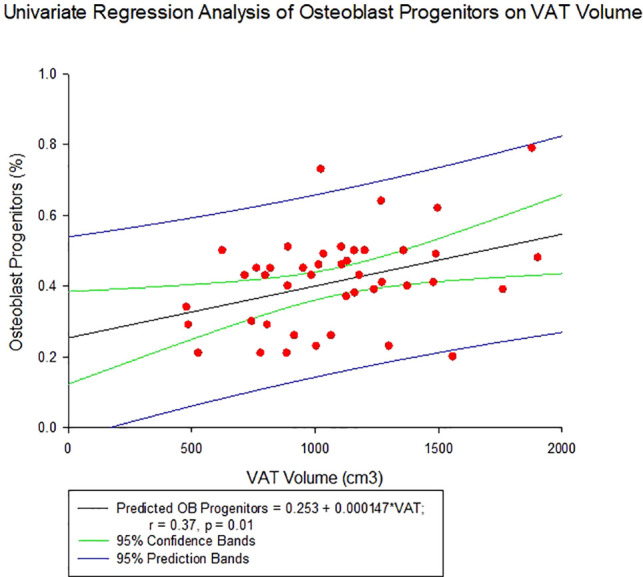
Regression of Circulating Osteogenic Progenitors (COP) with Visceral Adipose Tissue Volume. COP were identified as CD3-CD19-CD56-and OCN+ cells, and expressed as a percent of the nucleated, viable population.

Average total body fat percentage was 34.3 ± 4.3%. COP increased significantly with increasing VAT (r = 0.37, p = 0.01, see **Table 4** and **Figure 2**). There was no significant correlation between any of the OCPs with any parameter of body composition.

**Table 4:** Correlation analysis of body composition parameters on circulating osteogenic progenitors and osteoclast precursors.

Parameter	COP (n = 45)		CD14CD11b+(n = 45)		CD14MCSFR+(n = 44)		CD14CD120b (n = 45)	
	r	(p)	r	(p)	r	(p)	r	(p)
VAT volume (cm^3^)	0.37	**(0.01)**	0.01	(0.96)	-0.003	(0.98)	0.06	(0.69)
Body Fat (%)	-0.01	(0.95)	0.02	(0.89)	0.03	(0.86)	0.09	(0.57)
ALM (g)	0.19	(0.23)	0.22	(0.16)	0.18	(0.26)	0.27	(0.08)
Lean Mass (g)	0.18	(0.24)	0.18	(0.25)	0.15	(0.34)	0.22	(0.15)
L Spine BMD (g/cm^2^)	0.02	(0.90)	0.31	**(0.04)**	0.27	(0.08)	0.28	(0.06)
Femoral Neck BMD (g/cm^2^)	-0.06	(0.68)	0.03	(0.85)	0.05	(0.74)	0.03	(0.85)
Total body BMD (g/cm^2^)	-0.22	(0.14)	0.08	(0.60)	0.10	(0.52)	0.05	(0.74)
TBS	-0.07	(0.63)	0.06	(0.68)	0.05	(0.73)	0.01	(0.93)

Bolded p-values are statistically significant. COP, circulating osteogenic progenitors; VAT, visceral adipose tissue; ALM, appendicular lean mass; L Spine, Lumbar spine; BMD, bone mineral density; TBS, trabecular bone score.

**Figure 3:**
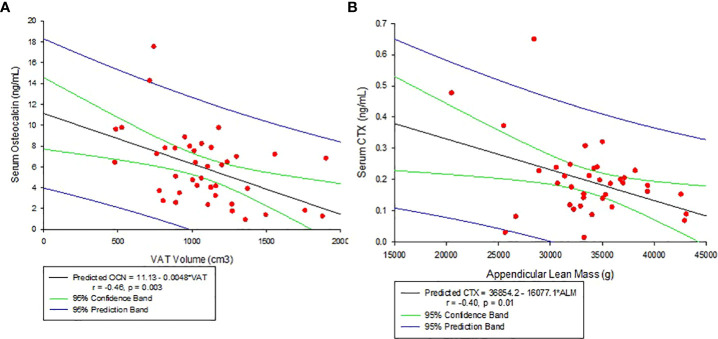
**(A)** Correlation between Serum Osteocalcin and Visceral Adipose Tissue Volume (VAT). **(B)** Correlation between Serum C-telopeptide (CTX) and Appendicular lean mass.

Other associations noted with body composition parameters are: VAT volume decreased significantly with increasing serum OCN levels (r = -0.46, p = 0.003, see **Figure 3A**) and negatively but weakly corelate with CTX (r = -0.31, p = 0.06). Appendicular lean mass, on the other hand, negatively correlated with CTX (r = -0.40, p = 0.01, see **Figure 3B**). There was no significant association between VAT volume and hs-CRP (r=0.22, p=0.12).

### Association between COP and OCPs with bone mineral density

There were no significant associations with bone density at the hip or femoral neck, but lumbar spine BMD was positively associated with all three OCP cell populations, though only the CD14CD11b+ reached statistical significance (r = 0.31, p = 0.04) (see **Table 4**).

### Independent predictors of circulating bone progenitor/precursor cells

Multivariable regression analyses were performed to identify the significant predictors of COP and OCP concentration in peripheral blood (**Table 5**). VAT volume and OCP concentration were found to be independent predictors for the COP (R^2 =^ 33.1, p < 0.001 for the model). For each of the CD14+CD11b+, CD14+MCSFR+, and CD14+CD120b+ cells, the independent predictors were found to be: age and COP; fasting plasma glucose and COP; 25-OH vitamin D and COP (R^2^ = 20.7, p = 0.007, R^2 =^ 27.4, p = 0.002, R^2 =^ 19.9, p = 0.008, respectively).

**Table 5:** Multivariate regression analysis of circulating osteogenic progenitors and osteoclast precursors with variables of interest.

Parameter	*R^2^*	β Estimate	SE	P	P (model)
***COP***	33.1				<0.001
CD14MCSFR+ (%)		0.031	0.010	0.005	
VAT Volume (cm^3^)		1.8×10^-4^	5.7×10^-5^	0.003	
***CD14CD11b+***	20.7				0.007
Age (years)		0.093	0.042	0.03	
COP (%)		4.46	1.96	0.03	
***CD14MCSFR+***	27.4				0.002
Glucose (mg/dL)		-0.010	0.004	0.02	
COP (%)		4.951	1.810	0.009	
***CD14CD120b+***	19.9				0.008
D-25OH (ng/mL)		0.048	0.021	0.03	
COP (%)		4.718	2.133	0.03	

R^2^ is expressed in percentage values; β Estimate is unitless. All significant variables from simple correlation analysis were included as candidate predictors. COP, circulating osteogenic progenitors; SE, standard error, VAT, visceral adipose tissue; D-25OH, 25-hydroxyvitamin D.

## Discussion

Our results show that in our population of mostly hypogonadal patients with T2DM, long-term poor glycemic control was associated with increased circulating COP compared to those with good long-term control. There were no significant differences in OCPs in either short- or long-term glycemic control status. COP positively correlated with VAT volume and negatively with free T. OCPs positively correlated with age, 25-hydroxyvitamin D, and FSH and negatively with plasma glucose, triglycerides, and free estradiol. Our results also showed that COP and OCP flux are positively correlated with each other as expected from the coupling mechanism. Further analysis revealed that VAT and OCPs are independent predictors of circulating COP; conversely, for OCPs independent predictors include COP for each, along with age; glucose; and 25-hydroxyvitamin D for the CD14CD11b+, CD14MCSFR+, and CD14CD120b+ cell populations respectively. Interestingly, we also found that OCN and CTX were inversely correlated with VAT and ALM respectively.

Osteoblasts are mononucleated cells whose primary function is to synthesize bone matrix. They derive from mesenchymal stem cells which are common progenitors for fibroblasts, chondrocytes, myoblasts, and adipocytes (32, 33). The process of differentiation into osteoblasts has been described in greater detail elsewhere (34–36), but briefly this common progenitor can be directed toward adipocytes by peroxisome proliferator-activated receptor gamma (PPARγ), myocytes by myoblast determination protein 1 (MyoD), chondrocytes by Sox-9, and osteoblasts by Runx-2. Upon Runx-2 activation, the cells undergo a 3-stage differentiation which results in a mature, osteocalcin (OCN)-secreting osteoblast (35).

Osteoclasts are multinucleated cells whose primary function in bone remodeling is to resorb bone. Mononuclear hematopoietic stem cells, in the presence of macrophage colony stimulating factor (M-CSF), differentiate into macrophage colony forming units, which are common precursors of both macrophages and osteoclasts. Receptor activator of nuclear factor κB (RANK) receptor activation by its ligand RANKL (among other downstream molecules) is then responsible for the differentiation of OCPs into mature osteoclasts (37–41). Multinucleation, stimulated by osteoclast stimulatory transmembrane protein (OC-STAMP) and dendritic cell-specific transmembrane protein (DC-STAMP) (42, 43), is a complex process which appears to strongly enhance resorptive activity (44). Mature osteoclasts resorb bone at their ruffled surface through acid secretion and proteolysis to dissolve the inorganic and organic components of bone respectively (45–47).

That osteoblast and osteoclast activities are coupled has been recognized for decades (48, 49). Bone remodeling occurs in four phases: activation, during which osteoclasts are recruited; resorption when osteoclasts are active, reversal when osteoclasts undergo apoptosis, and formation where osteoblasts lay down new bone (50). This coupling process is delicately regulated by a number of cell-cell communication mechanisms, and mediators (51–57). The crosstalk between these 2 cells is well-illustrated in our study by the positive correlation between COP and OCPs, and one an independent predictor of the other.

The potential role of COP in health (for example, healing of fractures) and diseases such as vascular calcifications, heterotopic ossification, osteoporosis, and frailty is summarized in a recent review by Feehan and colleagues (58). A study in a population of 57 older adults demonstrated a positive correlation between BMD and bone mineral content of the whole body and femoral neck with levels of COP (59), Interestingly, the authors identified that a COP cut-off of 0.4% has 100% sensitivity and 79% specificity of predicting osteoporosis on the femoral neck. Furthermore, they also found COP to positively correlate with appendicular lean mass (59). Moreover, with the emerging body of evidence suggesting that diabetes is associated with low bone turnover (60–62) some investigators examined the role of COP as reflection of ongoing events in the bone of patients with T2DM. In a study by Manavalan et al. (13), circulating OCN+ cells or COP cells are significantly lower in patients with T2DM compared to those without diabetes. This observation was corroborated by findings of significantly reduced bone turnover markers in the serum and significantly lower bone formation rate, osteoblastic surface, and osteoid surface by histomorphometric analysis of iliac crest biopsy of patients with T2DM compared to those without diabetes. Unfortunately, they did not quantify circulating OCPs in this study, although parameters of osteoclast activity and number in the bone were examined. Given the cross-talk between osteoblasts and osteoclasts with reduced bone formation, it is expected that reduction in bone resorption follows with resultant low bone turnover. In previous studies, our group also reported that men with T2DM with or without hypogonadism (2) and obese men with T2DM have reduced bone turnover compared to those without T2DM (29). Furthermore, we demonstrated in another study that glycemic control could be an important determinant of this alteration in bone turnover in T2DM patients, with poor glycemic control associated with reduction in bone turnover as compared to those with good glycemic control (7).

However, in the current study, we found the opposite of what we anticipated initially. Those with poor long-term glycemic control in fact had higher COP compared to those with good long-term glycemic control. A previous study reported an increase in OCN+ cells in the circulation of subjects with A1c in the pre-diabetic and diabetic range compared to normal subjects. The authors speculated that this increase in OCN+ cells may initiate or account for increased vascular calcification in these patients (28). In fact, one study reported that OCN+ cells are associated with the severity of aortic calcification (63). Lineage plasticity of the mesenchymal stem cells is influenced by a variety of factors (64). It is possible that poor metabolic health alters the balance between osteoblastogenesis and adipogenesis favoring the former over the latter as compensatory response to osteoblast apoptosis from hyperglycemia (65, 66). On the other hand, a published report indicated that hyperglycemia also retards maturation of osteoblast progenitor cells in a dose-dependent manner (11) and may also contribute to the high COP in those with poor glycemic control. The combined effect of hyperglycemia on the mature and differentiating osteoblastic cells is manifested clinically by dramatic reductions in bone formation indices histomorphometrically and on circulating COP followed by overall global reduction in bone turnover (13) (67–70). Although we did not find any concomitant increase in OCPs in our subjects, the segregation of the OCPs into different groups according to staining may have prevented us from finding a difference in these cells between the poor and good glycemic control groups.

As expected, there were significant negative correlations between the different OCPs with estradiol, and free T, deficiencies of which will result in increased bone turnover and bone loss (71–73). FSH receptors have been reported on the surface of osteoclasts (74), hence the finding of positive association between FSH and OCPs is not surprising. The negative relationship between OCPs and glucose, and triglycerides is likely a reflection of the suppression of bone remodeling that happens with T2DM and metabolic syndrome. However, we find an interesting positive correlation between COP and VAT, with the latter an independent predictor (in conjunction with OCPs) of the former in the multivariable regression analysis. One possible explanation for our observation could be the common progenitor shared by adipocytes and osteoblasts. Our cohort had average BMI of 31.7 and average total body fat of 34.3%, so they were more obese than the general population. It is possible that with more signals for this progenitor to differentiate into adipocytes, there was less signal to differentiate into osteoblasts. This is a possibility in patients with T2DM as hyperglycemia has been reported to divert OBP cells to an adipogenic pathway (75). Another possible explanation is VAT serving as a proxy for bone marrow adipose tissue (BMAT). Bredella et al. found a positive association between visceral fat and bone marrow fat, and additionally found an inverse correlation between marrow fat and trabecular BMD (76). Interestingly, when mesenchymal stem cell-derived osteoblasts are co-cultured with adipocytes, they show an increase in adipogenic and decrease in osteogenic markers (77). One study found that, compared to age-matched controls, people with osteoporosis have significantly more BMAT (78). Our findings also show that VAT is negatively associated with markers of bone formation (OCN), which in the presence of increased COP perhaps suggest that increased adipogenesis may suppress preosteoblast differentiation in our patient population, which would in turn decrease rate of bone remodeling and lead to poorer bone quality. Such findings have been suggested previously in the context of osteoporosis (79). We also found a negative association between CTX and ALM. One possible link between the bone homeostasis and muscle mass here could be RANKL’s activation of the NF- κB pathway and concomitant inhibition of myogenic differentiation (80, 81). It has been reported that treatment with denosumab, a RANKL inhibitor, can improve muscle mass in osteoporotic, sarcopenic mice (82). Similar observations have also been noted in older, community-dwelling adults (83).

We were also interested in learning whether COP and OCPs concentrations would be associated with BMD. We found a positive association between OCPs and lumbar spine BMD. Excessive mature OC activity can result in decreased bone mass (84, 85). Accordingly, our result could be interpreted as higher bone density caused by reduced bone turnover, a product of fewer OCP’s differentiating into mature osteoclasts. The positive correlation between OCPs and 25-hydroxyvitamin D, is consistent with the report from prior *in-vitro* studies showing that conversion of 25-hydroxyvitamin to 1,25-dihydroxyvitamin D occurs in macrophage lineage cells which results in increase in osteoclast transcription factors and regulation of osteoclastic differentiation (86, 87).

The strengths of this study include: 1) novelty, as this is the first study that examines the effect of glycemic control and other factors on the circulating bone precursor cells in T2DM and 2) the relatively good number participants for a flow cytometry study done on humans, which allows us to extrapolate a mechanistic understanding of previously observed trends in bone quality and geometry in patients with type 2 diabetes (2, 29). There are several limitations to our study. First, our patient population included mainly middle-aged men with T2DM, who for the most part are hypogonadal, and in the absence of normal controls and women, results from this study may not be applicable to the population of patients with T2DM in general. Second, although COP were higher in patients with Mean A1c>7%, the same trend was not observed for those with baseline A1c>7%, suggesting that the effect was either not strong, or that the effect of poor glycemic control on COP is a long-term relationship and may be difficult to capture at a single A1c timepoint. Finally, despite having significant p values, many of our correlation coefficients were somewhat weak.

In conclusion, findings from our study suggest high COP could be a marker for poor metabolic health with increased COP representing a potential compensatory response to the deleterious effect of hyperglycemia on osteoblasts (65, 66). However, there is also a possibility that poor metabolic health retards the maturation of COP cells to mature osteoblast (11). Given the different roles that COP may play in physiological and pathological conditions (58), future studies with larger sample size, longitudinal follow-up, and more demographically diverse patient population including women are needed to affirm results in this study.

## Data availability statement

Restrictions apply to the availability of some or all data generated or analyzed during this study to preserve patient confidentiality or because they were used under license. The corresponding author will on request detail the restrictions and any conditions under which access to some data may be provided.

## Ethics statement

This study involving human participants was conducted at the Michael E. DeBakey VA Medical Center in accordance with guidelines of the Declaration of Helsinki for the ethical treatment of human subjects. The protocol was approved by Baylor College of Medicine Internal Review Board. Each participant provided written informed consent to participate in this study.

## Author contributions

Research concept was conceived and funding secured by RA-V and DV. Data collection and sample analysis were performed by EB, VR, VF, MP and SB. Data analysis was conducted by EB, FD, CQ, and RA-V. Manuscript was written by EB, FD, and RA-V, and was edited by EB, FD, CQ, and RA-V. All authors contributed to the article and approved the submitted version.

## Funding

This work was supported by the US Department of Veterans Affairs Clinical Sciences Research and Development Merit Review Award 101CX001665 (to RAV) and National Institutes of Health (R01HD093047 to RAV).

## Conflict of interest

The authors declare that the research was conducted in the absence of any commercial or financial relationships that could be construed as a potential conflict of interest.

## Publisher’s note

All claims expressed in this article are solely those of the authors and do not necessarily represent those of their affiliated organizations, or those of the publisher, the editors and the reviewers. Any product that may be evaluated in this article, or claim that may be made by its manufacturer, is not guaranteed or endorsed by the publisher.

## Author Disclaimer

The contents do not represent the views of the U.S. Department of Veterans Affairs or the United States Government.
