# Supplementary material for: Circulating osteogenic progenitors and osteoclast precursors are associated with long-term glycemic control, sex steroids, and visceral adipose tissue in men with type 2 diabetes mellitus
Source: Front Endocrinol (Lausanne). 2022 Sep 12;13:936159. doi: 10.3389/fendo.2022.936159 (PMC9511027; doi:10.3389/fendo.2022.936159)
Supplement: Supplementary file 1 [file DataSheet_1.docx]

| **Supplementary Table 1. Participant Medications** | |
| --- | --- |
| **Regimen** | **Number of participants** |
| **No medication** | **9** |
| **On medications** | **42** |
| **Metformin only** | **8** |
| **Insulin only** | **1** |
| **Incretins only** | **1** |
| **Insulin+Metformin** | **4** |
| **Metformin+other non-insulin** | **11** |
| **Insulin+other non-Metformin** | **3** |
| **Insulin+Metformin+others** | **14** |
| Incretins include Dipeptidyl Peptidase 4 inhibitors and Glucagon-like peptide-1 receptor agonists. | |

***Supplementary Table 2****. Patient Characteristics According to A1c at Study Entry*

| **Parameter** | **A1c≤7% (n = 18)** | **A1c>7% (n = 33)** | **p-value** |
| --- | --- | --- | --- |
| Age (years) | 56.0 ± 5.9 | 54.8 ± 6.5 | 0.50 |
| Duration T2DM (years) | 5.0 ± 4.5 | 9.0 ± 5.9 | **0.02** |
| Avg A1c Prior Year (%) | 6.8 ± 1.0 | 9.0 ± 1.9 | **<0.001** |
| Glucose (mg/dL) | 131.5 ± 29.5 | 181.3 ± 69.7 | **0.006** |
| Triglycerides | 139.2 ± 89.1 | 325.0 ± 412.9 | 0.07 |
| HDL (mg/dL) | 40.6 ± 9.6 | 38.9 ± 12.8 | 0.62 |
| LDL (mg/dL) | 104.9 ± 49.1 | 124.7 ± 155.8 | 0.60 |
| 25-OHD (ng/mL) | 33.1 ± 14.7 | 23.8 ± 11.3 | **0.02** |
| LH (mIU/mL) | 5.7 ± 3.2 | 4.9 ± 2.2 | 0.30 |
| FSH (mIU/mL) | 14.4 ± 11.3 | 9.5 ± 8.4 | 0.09 |
| SHBG (nmol/L) | 30.5 ± 11.3 | 22.0 ± 12.6 | **0.02** |
| Testosterone (ng/dL) | 282.3 ± 93.6 | 255.2 ± 78.5 | 0.26 |
| Estradiol (pg/mL) | 18.9 ± 10.6 | 17.9 ± 7.8 | 0.71 |
| OCN (ng/mL) | 7.3 ± 4.0 | 5.2 ± 3.0 | 0.07 |
| CTX (ng/mL) | 0.20 ± 0.09 | 0.20 ± 0.14 | 0.92 |
| ALM (g) | 35321 ± 3216 | 33022 ± 4871 | 0.11 |
| Lean Mass (g) | 67546 ± 5771 | 63468 ± 8574 | 0.09 |
| VAT Volume (cm^3^) | 1133.9 ± 332.0 | 1064.5 ± 323.3 | 0.49 |
| Total Body Fat (%) | 35.0 ± 3.3 | 34.0 ± 4.3 | 0.43 |
| Lumbar Spine BMD (g/cm^2^) | 1.156 ± 0.21 | 1.122 ± 0.21 | 0.60 |
| Femoral Neck BMD (g/cm^2^)  Total Hip BMD (g/cm^2^) | 0.928 ± 0.17  1.159 ± 0.16 | 0.882 ± 0.13  1.062 ± 0.16 | 0.31  **0.05*** |
|  |  |  |  |

Results are expressed as mean ± standard deviation. Bolded p-values are statistically significant. Abbreviations: T2DM: type 2 diabetes mellitus, Avg: average, A1c: glycated hemoglobin, HDL: high-density lipoprotein, LDL: low-density lipoprotein, 25-OHD: 25-hydroxyvitamin D, LH: leutinizing hormone, FSH: follicle-stimulating hormone, SHBG: sex hormone binding globulin, OCN: osteocalcin; CTX: C-telopeptide of type I collagen; ALM: appendicular lean mass, VAT: visceral adipose tissue. *p = 0.14 after adjustment for age and BMI. A separate analysis for BMD and body composition adjusted for age and BMI showed no significant between-group differences in any of these parameters.

***Supplementary Table 3****. Patient Characteristic According to Mean -12 Months A1C21*

| **Parameter** | **A1c≤7% (n = 13)** | | **A1c>7% (n = 34)** | **p-value** |
| --- | --- | --- | --- | --- |
| Age (years) | | 57.0 ± 5.5 | 54.4 ± 6.5 | 0.19 |
| Duration T2DM (years) | | 4.7 ± 4.1 | 8.9 ± 5.9 | **0.02** |
| Glucose (mg/dL) | | 123.5 ± 25.3 | 180.2 ± 67.1 | **0.004** |
| Triglycerides | | 154.6 ± 173.6 | 299.4 ± 392.3 | 0.19 |
| HDL (mg/dL) | | 43.2 ± 9.5 | 38.2 ± 12.4 | 0.18 |
| LDL (mg/dL) | | 108.5 ± 52.2 | 121.2 ± 149.6 | 0.76 |
| D-25OH (ng/mL) | | 32.5 ± 16.7 | 24.9 ± 11.5 | 0.08 |
| LH (mIU/mL) | | 8.6 ± 10.8 | 4.9 ± 2.3 | 0.08 |
| FSH (mIU/mL) | | 14.1 ± 12.1 | 10.0 ± 8.5 | 0.18 |
| SHBG (nmol/L) | | 31.1 ± 12.3 | 22.9 ± 12.1 | **0.04** |
| Testosterone (ng/dL) | | 279.2 ± 107.3 | 262.9 ± 73.2 | 0.54 |
| Estradiol (pg/mL) | | 19.4 ± 12.2 | 18.0 ± 7.5 | 0.64 |
| OCN (ng/mL) | | 6.8 ± 4.7 | 5.7 ± 3.0 | 0.39 |
| CTX (ng/mL) | | 0.20 ± 0.06 | 0.20 ± 0.13 | 0.99 |
| ALM (kg) | | 35.3 ± 2.8 | 32.9 ± 4.6 | 0.09 |
| Lean Mass (kg) | | 68.2 ± 5.1 | 63.1 ± 8.1 | **0.04*** |
| VAT Volume (cm^3^) | | 1144 ± 353 | 1043 ± 288 | 0.32 |
| Total Body Fat (%) | | 35.2 ± 3.6 | 33.9 ± 4.6 | 0.36 |
| Lumbar Spine BMD (g/cm^2^) | | 1.178 ± 0.20 | 1.107 ± 0.21 | 0.28 |
| Femoral Neck (g/cm^2^) | | 0.941 ± 0.18 | 0.875 ± 0.13 | 0.16 |
| Total Hip (g/cm^2^) | | 1.155 ± 0.16 | 1.063 ± 0.16 | 0.08 |
|  | |  |  |  |

Results are expressed as mean ± standard deviation. Bolded p-values are statistically significant. Abbreviations: T2DM: type 2 diabetes mellitus, HDL: high-density lipoprotein, LDL: low-density lipoprotein, A1c: glycated hemoglobin, D-25OH: 25-hydroxyvitamin D, LH: leutinizing hormone, FSH: follicle-stimulating hormone, SHBG: sex hormone binding globulin, OCN: osteocalcin; CTX: C-telopeptide of type I collagen; ALM: appendicular lean mass, VAT: visceral adipose tissue. *p=0.20 after adjustments for age and BMI. A separate analysis for BMD and body composition adjusted for age and BMI showed no significant between-group differences in any of these parameters.
